# More Evidence about Monkeypox Sexual Transmission in the Current 2022 Multi-Country Outbreak. Reply to Vera et al. Comment on “Sah et al. Monkeypox and Its Possible Sexual Transmission: Where Are We Now with Its Evidence? *Pathogens* 2022, *11*, 924”

**DOI:** 10.3390/pathogens11121418

**Published:** 2022-11-25

**Authors:** Abdullah Reda, Ranjit Sah, Alfonso J. Rodríguez-Morales

**Affiliations:** 1 Faculty of Medicine, Al-Azhar University, Cairo 11651, Egypt; 2 Tribhuvan University Teaching Hospital, Institute of Medicine, Kathmandu 44600, Nepal; 3 Department of Global Health and Clinical Research, Harvard Medical School, Boston, MA 02115, USA; 4 Department of Microbiology, D.Y Patil Medical College, Hospital and Research Centre, Dr. D.Y. Patil Vidyapeeth, Pune 411018, Maharashtra, India; 5 Grupo de Investigación Biomedicina, Faculty of Medicine, Fundacion Universitaria Autonoma de las Americas, Pereira 660001, Risaralda, Colombia; 6 Master of Clinical Epidemiology and Biostatistics, Universidad Cientifica del Sur, Lima 15023, Peru; 7 Gilbert and Rose-Marie Chagoury School of Medicine, Lebanese American University, Beirut P.O. Box 36, Lebanon

We want to thank Milagros N. Vera and colleagues [1] for their exciting comments about the sexual transmission of monkeypox (MPX) at their centre in Peru. As it has been widely reported in the current MPX outbreak, the authors further demonstrated that most of the reported cases at their centre were related to sexual activities, which we also showed in our literature review about the sexual transmission of MPX [2]. Indeed, we have conducted some related reports that further indicated the sexual transmission of MPX in the current outbreak. For instance, in a systematic review of the epidemiology of MPX, León-Figueroa et al. [3] showed that 3876 of 4222 MPX-confirmed cases resulted from recent sexual contact. Furthermore, it has been further found that lesions were most frequently located in the perianal, genital, and oral regions, further proving that MPX is transmitted by sexual contact.

It can be argued that MPX sexual transmission is not necessarily a sexually transmitted disease since the MPX virus (MPXV) has been historically known to transmit through close contact; the current claims about such events are novel and specific to the recent outbreak and were not, or meagrely, reported in previous ones. That might be true since no current evidence can adequately explain the mechanism of the current distribution of the locations of MPX lesions. However, in the meta-analysis by Reda et al. [4], we found that 72.4% of MPX-confirmed cases have the virus in their seminal specimens. Although the replication-competence of the viral particles detected in these specimens remains controversial [4,5], the high rate of MPXV detection in semen strongly suggests its sexual transmission. Moreover, various reports have indicated that the current outbreak has affected chiefly male individuals, especially those who have sex with other men (MSM) [2,6], adding further evidence that the virus can be sexually transmitted. However, it should be noted that the disease was also detected among women. For instance, Thornhill et al. [7] recruited 120 infected women across 15 countries, including 56 transgender, 64 cis women, and five non-binary individuals assigned females at birth. The authors demonstrated that MPX infection was transmitted sexually in 61% and 77% of cis and trans women, respectively.

Moreover, 78% of the lesions were in the anogenital region. Finally, MPXV could be detected in 100% (7/7) of the tested vaginal samples. Accordingly, it can be concluded that MPXV can be sexually transmitted and is attributed to many infections in the current 2022 outbreak.

One additional point to consider is the significant prevalence of the infection among MSM. Although the disease is highly prevalent among this population, this should not be stigmatized, and proper care and interventions should be adequately offered to reduce viral transmission [8]. Moreover, the recent report by Thornhill et al. [7] showed that 85% of their female population with MPX reported having sex with men. That further proves that the disease is sexually transmitted and, most importantly, is not exclusive to MSM and other LGBTQ+ communities. Accordingly, worldwide efforts should be directed to enhance interventional approaches for curbing the sexual transmission of MPXV among different population groups, without unnecessary discrimination.
